# Supplementary material for: Complexome profiling of the Chlamydomonas psb28 mutant reveals TEF5 as an early PSII assembly factor
Source: Plant Cell. 2025 Mar 17;37(6):koaf055. doi: 10.1093/plcell/koaf055 (PMC12164590; doi:10.1093/plcell/koaf055)
Supplement: koaf055_Supplementary_Data [file koaf055_supplementary_data.zip › psb28_interactive.zip!/plots/intensity/qualityPlots/peptides/_Cre11.g467682.t1.1.html]

### Cre11.g467682.t1.1

Trivial name:   
SeqCoverage [median]: 9.60  
Mass: 37.66 kDa  
  
 

| Sequence | MissedCleavage | Mass [Da] | wtIntensity | muIntensity | Score |
| --- | --- | --- | --- | --- | --- |
| AAAAAAAEAGGIVAR | 0 | 1268.68 | 1406.34 | 475.98 | 147.91 |
| QAGAGVESVGR | 0 | 1029.52 | 337.86 | 395.26 | 67.73 |
|  |  |  | 1744.2 | 871.2 |  |
